# Adults with spina bifida: A cross‐sectional study of health issues and living conditions

**DOI:** 10.1002/brb3.1736

**Published:** 2020-07-07

**Authors:** Martina Bendt, Hanna Gabrielsson, Dorothee Riedel, Göran Hagman, Claes Hultling, Erika Franzén, Mats Eriksson, Åke Seiger

**Affiliations:** ^1^ Rehab Station Stockholm Spinalis Outpatient Clinic Solna Sweden; ^2^ Department of Neurobiology, Care Science and Society Karolinska Institutet Stockholm Sweden; ^3^ Faculty of Medicine and Health School of Health Sciences Örebro University Örebro Sweden; ^4^ Ersta Sköndal Bräcke University College Stockholm Sweden; ^5^ Spinalis Foundation Stockholm Sweden; ^6^ Department of Neurobiology, Care Science and Society Division of Physiotherapy Karolinska Institutet Stockholm Sweden; ^7^ Function Area Occupational Therapy & Physiotherapy Karolinska University Hospital Stockholm Sweden; ^8^ Stockholms Sjukhem R&D Unit Stockholm Sweden

**Keywords:** adult, health issues, living conditions, myelomeningocele, spina bifida

## Abstract

**Objective:**

To describe health issues and living conditions in a cohort of adults living with Spina bifida.

**Material and methods:**

A cross‐sectional study was conducted by a multidisciplinary team.

Adults with spina bifida (*n* = 219) were invited to participate. One‐hundred‐and‐ninety‐six persons (104 women and 92 men; 18–73 years, median age 33 years) were included. Structured interviews, questionnaires, and clinical assessments for medical, social, physical, and cognitive functions were used.

**Results:**

There was large variation among participants as regards the consequences of their spina bifida. Individuals < 46 years seemed to have more secondary conditions such as hydrocephalus, Chiari II malformation, tethered cord symptoms, and latex allergy. A higher proportion of the individuals >46 years and older was able to walk, and they had performed better in primary school and on tests of psychomotor speed and executive function.

**Conclusions:**

This study demonstrates that adults with spina bifida have a complex set of physical, cognitive, and social needs that need to be addressed in order to improve their health issues and living conditions. The high prevalence of urinary and fecal incontinence, pain, and overweight underline that these issues need much attention during follow‐up. The future generations of older adults may need more attention in many ways, since they at a younger age do have more complex medical conditions, lower physical and cognitive functions, and lower prerequisites for independent living and participation in society than those > 46 years today. This elucidates that adults with spina bifida need systematic follow‐up services and social support throughout life.

## Graphical abstract

The aim was to describe health issues and living conditions of adults with Spina bifida. Today, persons who have survived to the age of >46 years had less complex medical conditions, better physical and cognitive functions, and had attained a higher level of education, leading to better prerequisites for living independently and participating in society. The future generations of older adults may need more attention in many ways, since they at a younger age do have more complex medical conditions, lower physical and cognitive functions, and lower prerequisites for independent living and participation in society than those> 46 years today. This elucidates that adults with Spina bifida need systematic follow‐up services and social support throughout life.

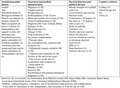

## 1 INTRODUCTION

Spina bifida (SB) is a congenital spinal neural tube defect (NTD) (McComb, 2015) often used synonymously with myelomeningocele (MMC) (Alriksson‐Schmidt, Josenby, Lindquist, & Westbom, 2018) involving multiple body systems (Bakaniene, Ziukiene, Vasiliauskiene, & Prasauskiene, 2018; Wagner et al., 2015). The annual incidence of SB in Sweden has decreased over the last decade to 1.3 per 10,000 newborns/year (Bodin et al., 2018) and is now considered to be a rare condition in the country. However, the total prevalence group has probably not decreased since their life expectancy has increased, thanks to the development of medical treatments. Today, the majority of children born with SB are expected to live into adulthood (Dicianno et al., 2008; Olsson et al., 2007). A larger population of adults with SB will most likely need more attention in the future, indicating a need for extended knowledge of the adult population.

In SB, the degree of sensory and motor impairment depends on the level and extent of spinal involvement, resulting in a complex disorder that affects many systems of the body at the same time, such as neurological, urological (including kidney/renal function), and musculoskeletal functions (Bakaniene et al., 2018). The presence of hydrocephalus (HC), Chiari II malformation, and other associated brain malformations also affect the severity of the outcome for the patient (Copp et al., 2015; Treble, Juranek, Stuebing, Dennis, & Fletcher, 2013). Comorbidities and secondary conditions are common (Alriksson‐Schmidt et al., 2013), such as disturbed sleep pattern (Murray et al., 2016), latex allergy (Bowman, McLone, Grant, Tomita, & Ito, 2001), pressure ulcers (Wagner et al., 2015), overweight (Polfuss, Bandini, & Sawin, 2017), tethered cord symptoms, and contractures in the lower extremities (Bakaniene et al., 2018; Copp et al., 2015).

Medical follow‐up during childhood is thoroughly monitored, whereas follow‐up in adulthood is often inconsistent or absent (Dicianno et al., 2008). Adults with SB have greater medical needs (Dicianno & Wilson, 2010) and significantly more hospitalizations per year than the general population (Dicianno et al., 2008; Dicianno & Wilson, 2010). Shunted HC is common (Wetzel, Heaner, Gabel, Tubbs, & Chern, 2018) and correlates with cognitive impairments (Hampton et al., 2011), which mainly affect the executive functions (Zabel et al., 2011) associated with the ability to be independent. The cognitive impairments become more evident in adolescence and adulthood, when expectations increase on independent living, a higher level of education, work, and relations (Dennis, Landry, Barnes, & Fletcher, 2006). Adulthood means higher demands not only on individuals with SB but also on their relatives and the social welfare and healthcare systems, as this group has a persistent need for care and support.

Up until now, most studies concerning adults with SB have focused on young adults (Young, Anselmo, Burke, McCormick, & Mukherjee, 2014), and the studies often describe specific impairments and limitations such as urological (Lindehall et al., 2008), neurological (Young et al., 2014), orthopedic (Wright, 2011), and neuropsychiatric issues (Zabel et al., 2011). A multidisciplinary approach to study the health and living conditions of adults with SB, including the aging population, is of importance since there is limited knowledge of the SB natural history (Bakaniene et al., 2018; Fletcher & Brei, 2010; Lidal, Lundberg Larsen, & Hoff, 2019; Webb, 2010).

This study is part of a larger research project aimed at describing a regional cohort of adults living with SB enrolled at a specialized outpatient clinic for adults with spinal cord disorders. The long‐term goal of the larger project is to target interventions to needs, and to increase knowledge of health issues and living conditions for adults with SB. The aim of the present study was to describe health issues and living conditions in a cohort of adults with SB.

## 2 MATERIAL AND METHODS

A cross‐sectional, population‐based study of adults (≥18 years) with SB (with neurological impact) living within the Greater Stockholm area was conducted. The research group comprised a multidisciplinary team, consisting of a physician, a nurse, a physiotherapist, a neuropsychologist, an occupational therapist, and a social worker.

Included were persons with myelomeningocele (MMC), lipomeningocele, and spina bifida occulta with neurological impairment.

### 2.1 Setting and participants

The Spinalis outpatient clinic is, since the last 20 years, responsible for healthcare follow‐ups for adults with SB in the Greater Stockholm area. Individuals 18 years and over with SB (*n* = 219), registered at this specialized outpatient clinic for adults with spinal cord disorders, were invited to participate.

### 2.2 Procedure

Data were collected during individual face‐to‐face sessions through interviews based on questionnaires and clinical assessments. The data collection was done in conjunction with the regular follow‐up based on the usual structure, however, extended and more systemized. All patients attending their regular follow‐up were asked for participation and informed verbally and in writing about the study. Those who accepted participation gave their signed informed consent, and those who declined received the usual structure on follow‐up. Medical records (including records from the children's hospital, computerized medical records, and paper records, depending on availability) were used to validate the information given. The interview included sociodemographic factors, medical information, and associated conditions. Due to cognitive impairment, some participants had help from their assistance provider to accept participation as well as answering the questions, in some cases, mainly for confirmation and in some cases with a major part of the questions.

An overview of the data collection and methods used is presented under four headlines in Table 1. The data collection methods are presented with assessment (A), interview (I), and information from medical records (M).

**TABLE 1:** Overview data collection and methods

Sociodemographic factors	Structural and medical characteristics	Physical function and assistive devices	Cognitive subtests
Gender Age Household status (I) Biological children (I) Passed core subjects in primary school (I) Main occupation (I) Driver's license (I) Independence and support in daily life (personal care, household activities, and/or reminders) (I) Economy guidance (I) Assistance provider (municipal/state and/or family members) (I) Transportation services (I)	Diagnosis (I + M) Length (cm) (A) Weight (kg) (A) Hydrocephalus (I + M) Yes/no Shunt and number of revisions (I + M) Chiari II malformation (I + M) Diagnosis (M) Type of Spina Bifida Epilepsy (I + M) Blood pressure (A) Allergy (I + M) Drug treatment (I + M) Secondary complications ‐ Pressure ulcer ‐ (on examination day, presence, and location) (A) ‐ Orthopedic surgery related to SB (I + M) ‐ Tethered cord symptoms (I + M) (Earlier in life and/or last year) ‐ Pain (yes/no on the day of examination and location) (I) ‐ Sleep habits (I) Bladder^a^ (I + M) Bowel (I + M) Psychological and neuropsychological diagnoses (I + M)	Muscle strength (0–5 graded scale) (A) Level of motor and sensory function (AIS) Contractures > 20 degrees in hip, knee or > 15 degrees ankle joints (A) Mode of mobility Transfers^b^ (A) Weekly physical exercise habits (No physical exercise, moderate exercise or strenuous physical exercise) (I) FIM (A + I) Hand function (A) Assistive devices (A + I)	‐ Coding (A) ‐ Block design test (A) ‐ FAS (A)

Note

Interview (I), Assessment (A), Information from Medical records (M), Spina bifida (SB), American Spinal Injury Association Impairment Scale (AIS), Functional Independence Measure (FIM).

^a^ Method, voiding frequency, continence, use of incontinence pads and size, and complications.

^b^ From chair or wheelchair to bed. Independent, with assistance or with the use of a lift.

#### 2.2.1 Sociodemographic factors

Data were collected through structured interviews; see Table 1.

#### 2.2.2 Structural and medical characteristics

Data were collected through structured interviews, assessments, and information from medical records; see Table 1. Height in standing position or length (for those not able to stand) in the prone position (from joint to joint in the event of contractures) and weight were measured, and body mass index (BMI; kg/m^2^) was calculated (WHO, 2017a).

Bladder and bowel regimes were assessed with NSCIR (Levi & Ertzgaard, 1998), and part of the results are presented in Table 3. More in‐depth results will be presented in a separate article (Ehrén, Lindbo, Gabrielsson, Bendt, & Seiger, 2020 accepted for publication).

Neuropsychological diagnoses (Asperger syndrome, attention deficit disorder, attention deficit hyperactivity disorder), psychological diagnoses (depression and anxiety), and intellectual disability were collected from medical records and interview. Self‐reported sleep habits were registered according to a modified questionnaire based on the Basic Nordic Sleep Questionnaire (Partinen & Gislason, 1995) and were categorized as difficulty falling asleep, waking up in the middle of the night and not being able to go back to sleep, or sleeping too long.

#### 2.2.3 Physical function and assistive devices

The participants were assigned to different muscle function (MF) groups classified as I–V (Bartonek, Saraste, & Knutson, 1999) with the additional classification MF 0 (Bendt & Bartonek, 2016). MF 0 represents those with no loss of muscle strength, and MF V represents those with no muscle activity in the lower limbs and no pelvic elevation, based on a muscle strength examination of the lower extremities (Hislop, 1995). AIS (Kirshblum et al., 2011) was used to determine the level of lesion, sensory/motor function. Contractures (>20 degrees) in hips, knees, or ankle joints (>15 degrees) were registered (American Academy of Orthopedic & Surgeons, 1988). Functional ambulation was registered according to the criteria by Hoffer et al (Hoffer, Feiwell, Perry, Perry, & Bonnett, 1973). The motor function part of the Functional Independence Measure (FIM) was used to measure the level of each participant's disability and to indicate how much assistance is required to carry out activities of daily living (Dodds, Martin, Stolov, & Deyo, 1993). Dexterity was assessed with the Nine‐Hole Peg Test (Mathiowetz, Weber, Kashman, & Volland, 1985), and hand strength was assessed with Grippit (Nilsen et al., 2012). Weekly physical exercise habits were registered based on the participants' own statements: no physical exercise, moderate exercise (a minimum of 30 min, 1–2 times weekly), and strenuous physical exercise (a minimum of 30 min at least three times weekly).

#### 2.2.4 Cognitive subtests

The coding test measures associative memory, graphomotor speed, and processing speed; the block design test measures visual spatial processing, visual motor construction, and problem solving (Wechsler, 2002); the FAS verbal fluency test measures verbal executive ability (Tallberg, Ivachova, Jones Tingheg, & Ostberg, 2008).

Data are presented for the total cohort and four age groups (Group 1: 18–30, Group 2: 31–45, Group 3: 46–60, and Group 4: ≥61 years) according to the recommended standardization for reporting of the International Spinal Cord Injury Core Data Set (DeVivo, Biering‐Sorensen, New, & Chen, 2011). The cognitive subtests are presented correspondingly and are assigned to persons with and without HC. The hand function parameters are divided into persons with and without HC.

### 2.3 Statistical analyses

Descriptive data are presented as numbers and proportions. The Shapiro–Wilk test was used to analyze normal distribution. Mean and standard deviation (*SD*) were used for normally distributed variables: median and interquartile ranges (IQRs); min–max values were used for non‐normal distributions. Differences between the groups and the presence or absence of HC were analyzed among the variables for cognition and dexterity using the chi‐square test for dichotomous variables, the Student *t* test for variables with normal distributions, and the Mann–Whitney *U* test for variables with non‐normal distributions. Statistical significance was determined to *p* ≤ .001. Statistically significant differences are presented in the results, except for those who accepted or declined participation in the cognitive tests. The analyses were performed using SPSS version 24 (IBM Corp.).

### 2.4 Ethical approval

The study was approved by the Regional Ethical Review Board in Stockholm (Dnr: 2014/1111‐31).

## 3 RESULTS

One‐hundred‐and‐ninety‐six persons (89%) (104 women and 92 men) were included; 19 declined participation in the study, and four did not respond despite repeated invitations. One‐hundred‐and‐fifty‐three persons (70%) (80 women and 73 men) underwent the cognitive assessment. There were no significant differences in age, gender, or prevalence of HC between those who performed the cognitive assessment and those who declined.

### 3.1 Sociodemographic factors

The participants had a median age of 33 years (IQR 23–46, min–max 18–73), 37 (IQR 23–50, min–max 18–73) years for women, and 30 (IQR 24–40, min–max 18–65) years for men with a significantly higher proportion of women in the two older groups (>46 years; *p* = .001). The participants were born between 1942 and 1997.

Fifty participants in Group I (56%) had passed core subjects in primary school, which was significantly lower (*p* < .001) than for the other groups. One‐hundred‐eleven participants (57%) stated that they had a driver's license, but only half of them (49%) drove on a regular basis, and 72% were entitled to transportation service. Seventy‐one % had assistance in daily life (with household activities and/or personal care and/or reminders). The assistance was provided by relatives (31%), and municipality and/or state agencies 44% (of whom 48% received extra help from relatives). Forty‐seven % received guidance concerning their personal economy. The sociodemographic factors are presented in Table 2.

**TABLE 2:** Sociodemographic factors

	Total *n* (%)	Age group 1 (18–30 years) *n* (%)	Age group 2 (31–45 years) *n* (%)	Age group 3 (46–60 years) *n* (%)	Age group 4 (≥61 years) *n* (%)
Participants	196	90 (46)	60 (31)	38 (19)	8 (4)
Gender					
Women	104 (53)	42 (47)	28 (47)	28 (74)	6 (75)
Men	92 (47)	48 (53)	32 (53)	10 (26)	2 (25)
Household status					
With partner*	35 (18)	4 (4)	15 (25)	13 (35)	3 (37.5)
Single living with child/children	6 (3)	—	4 (7)	2 (5)	—
With parents/friend	62 (31)	60 (67)	2 (3)	—	—
Single household	80 (41)	20 (22)	36 (60)	21 (55)	3 (37.5)
Sheltered housing	13 (7)	6 (7)	3 (5)	2 (5)	2 (25)
Biological children	32 (16)	1 (1)	9 (15)	17 (45)	5 (63)
Passed core subjects in primary school	135 (69)	50 (56)	46 (77)	30 (81)	8 (100)
Main occupation					
School/education	39 (20)	37 (41)	2 (3)	—	—
Unemployed	37 (19)	26 (29)	7 (12)	3 (8)	1 (12)
Protected workshop	15 (8)	6 (7)	7 (12)	2 (5)	—
Part‐time work	30 (15)	7 (8)	13 (21.5)	10 (26)	—
Full‐time work	43 (22)	14 (15)	18 (30)	8 (21)	3 (38)
Pensioner	32 (16)	—	13 (21.5)	15 (40)	4 (50)

Note

Number (*n*), * with or without children.

### 3.2 Structural and medical characteristics

A majority, 179 participants (91%), were diagnosed with MMC (35 thoracic, one thoracolumbar, 114 lumbar, eight lumbosacral, and 21 sacral level), 14 were diagnosed with lipomeningocele (one thoracic, six lumbar, three lumbosacral, and four sacral), and three in the cohort were diagnosed with spina bifida occulta (with neurological impact). Sixty‐three percent of the total cohort had HC. The younger participants, Group 1 (18–30 years), had a significantly higher prevalence of HC than the other groups (*p* < .001).

Fifty‐nine participants (30%) had a blood pressure (BP) below 110/70 mmHg, and 22 participants (11%) had a blood pressure over 140/80 mmHg. Thirty‐nine participants in Group 1 (18–30 years) (43%) reported that they had previously had, or still had, tethered cord symptoms, which is a significantly higher proportion than in the total cohort (*p* < .001). Of the 78 persons (40%) who experienced pain on the day of examination, 27 (35%) reported back pain, and 29 (37%) reported load‐related pain in the legs. Other locations of pain were head, shoulders, or arms. The most common location of pressure ulcers was the feet, followed by the sitting area.

Nine participants of the total cohort (4%) were underweight according to the BMI classification, 64 (33%) had a normal BMI, 64 (33%) were overweight, and 59 (30%) were obese. Structural and medical characteristics are presented in Table 3.

**TABLE 3:** Structural and medical characteristics

	Total *n* = 196	Age group 1 (18–30 years) *n* = 90	Age group 2 (31–45 years) *n* = 60	Age group 3 (46–60 years) *n* = 38	Age group 4 (≥61 years) *n* = 8
Diagnosis, *n* (%)					
MMC	179 (91)	84 (93)	56 (93)	32 (84)	7 (88)
Lipomeningocele	14 (7)	4 (4)	3 (5)	6 (16)	1 (12)
SB occulta	3 (1)	2 (2)	1 (2)	—	—
Hydrocephalus, *n* (%)	123 (63)	68 (76)	42 (70)	12 (32)	1 (13)
>2 shunt revisions, *n* (%)	48 (39)	19 (28)	19 (45)	10 (83)	—
Orthopedic surgery					
Earlier in life, *n* (%)	134 (68)	63 (70)	43 (72)	23 (61)	5 (63)
Last year, *n* (%)	2 (1)	2 (2)	—	—	—
Spinal fusion, *n* (%)	32 (16)	20 (22)	11 (18)	1 (3)	—
Tethered cord symptoms					
Earlier in life,* n* (%)	53 (27)	36 (40)	11 (18)	5 (13)	1 (12)
Last year, *n* (%)	5 (3)	3 (3)	2 (3)	—	—
Pain, *n* (%)	78 (40)	31 (34)	22 (37)	20 (53)	5 (63)
Epilepsy, *n* (%)	16 (8)	3 (3)	8 (13)	4 (11)	1 (13)
Latex allergy, *n* (%)	62 (32)	36 (40)	23 (38)	3 (8)	—
Pressure ulcers, *n* (%)	39 (20)	18 (20)	9 (15)	10 (26)	2 (25)
Length, mean (*SD*)	158 (14.1)	159 (14.4)	157 (13.5)	156 (13.7)	158 (17.3)
Women, mean (*SD*)	152 (12.3)	153 (13.5)	151 (12.5)	151 (10.7)	150 (11.9)
Men, mean (*SD*)	164 (13.2)	163 (13.5)	163 (11.9)	168 (14.4)	179 (12.7)
Weight, md (IQR)	67 (56–81)	65 (52–75)	71 (60–83)	67 (58–82)	66 (55–91)
Women, md (IQR)	63 (50–75)	55 (47–75)	66 (57–78)^a^	64 (53–73)^a^	60 (54–74)^a^
Men, md (IQR)	72 (61–85)	67 (60–82)	75 (68–84)^a^	88 (72–101)^a^	90 (87–90)^a^
BMI, md (IQR)	27 (23–31)	25 (16–52)	28 (20–45)	26 (21–59)	28 (18–36)
Women, md (IQR)	26 (22–32)	23 (21–28)	29 (25–35)^a^	26 (24–35)	28 (25–35)
Men, md (IQR)	27 (24–31)	26 (22–30)	28 (24–32)^a^	28 (25–33)	28 (25–28)
Bladder emptying method					
Spontaneous micturation^b^	40 (20)	14 (16)	9 (15)	13 (34)	4 (50)
CIC^c^	105 (54)	61 (67)	34 (57)	10 (26)	2 (25)
Indwelling catheter^d^	6 (3)	1 (1)	1 (2)	3 (8)	1 (12.5)
Urological surgery^e^	45 (23)	14 (16)	16 (26)	12 (32)	1 (12.5)
Bladder incontinence^f^	152 (78)	69 (76)	50 (83)	28 (74)	5 (63)
Bowel regime					
Normal^g^	123 (63)	48 (53)	38 (63)	30 (79)	8 (100)
Other regimes^h^	73 (37)	42 (47)	22 (37)	8 (21)	—
Faecal incontinence^f^	150 (77)	66 (73)	52 (86)	28 (74)	4 (50)

Note

Number (*n*), Myelomeningocele (MMC), Spina bifida (SB), Median (Md), (IQR) Interquartile range (IQR), Standard

Deviation (*SD*), Body Mass Index (BMI), Clean intermittent catheterization (CIC).

Normal distributions reported in mean, non‐normal reported in median.

^a^ Normally distributed, median used to compare with the groups not normally distributed.

^b^ Including strain.

^c^ Clean intermittent catheterization.

^d^ Kathéter À Demeure or supra pubic catheter.

^e^ Vesicostomy, continent urinary diversion or ileal conduit.

^f^ Ranging from daily ≤ once a month.

^g^ Including medication.

^h^ Reflex/straining, manual evacuation, ostomy, irrigation.

Bladder and bowel functions and distribution among the groups are reported in Table 3. Twenty‐four participants (12%) needed a reminder or practical help from another person to manage emptying their bladder. Disturbed sleep was reported by 122 participants (62%).

Reports suggested that 12 participants (6%) had neuropsychological diagnoses, 33 (17%) had psychiatric diagnoses, and 27 (14%) had intellectual disabilities.

### 3.3 Physical function and assistive devices

The most common neurological impairment level was at L3 (midlumbar), evident in 80 participants (41%). Contractures of more than 20 degrees in the hip, knee, and/or ankle joints (>15 degrees) were present in 165 participants (84%).

Functional ambulatory ability was distributed almost equally among Groups 1, 2, and 3 (i.e., one‐third reported that they always walked, one‐third reported that they always used a wheelchair, and one‐third combined walking with a wheelchair). Seven of the eight participants in Group 4 reported that they always walked. Transfer ability was similar among Groups 1, 2, and 3, with approximately 85% performing independent transfers from a chair or wheelchair to their bed. All participants in Group 4 performed independent transfers. Half of the group did not exercise on a regular basis; 40% reported a moderate level of physical exercise, and 10% performed strenuous physical exercise regularly.

Almost two‐thirds of the participants (121) reached full independence, or modified independence, according to the motor function part of FIM (>78 points) (the mean value for the entire group was 73, *SD* 19). Individuals with HC performed significantly lower on the dexterity assessment than the participants without HC (*p* < .001). Participants without HC scored below the reference values for the general population, and participants with HC scored three or more *SD* below the values for the general population. Hand strength was less impaired; participants without HC preformed within or slightly under reference values, and participants with HC performed one *SD* under reference values. Physical function characteristics and the use of assistive devices are reported in Table 4.

**TABLE 4:** Physical function characteristics and assistive devices

	Total *n* = 196 *n* (%)	Age group 1, (18–30 years) *n* = 90 *n* (%)	Age group 2, (31–45 years) *n* = 60 *n* (%)	Age group 3, (46–60 years) *n* = 38 *n* (%)	Age group 4, (≥61 years) *n* = 8 *n* (%)
Neurological category					
T3‐T12 AIS A	26 (13)	12 (13)	10 (16)	4 (11)	—
T3‐T12 AIS A ZPP	23 (12)	15 (17)	6 (10)	2 (5)	—
T3‐T12 AIS B, C, D	2 (1)	2 (2)	—	—	—
L1‐L2 AIS A	9 (5)	4 (4)	1 (2)	4 (11)	—
L1‐L2 AIS A ZPP	25 (13)	5 (6)	13 (22)	6 (15)	1 (13)
L1‐L2 AIS B, C, D	6 (2)	4 (4)	—	1 (2)	1 (13)
L3 AIS A	20 (10)	9 (10)	7 (12)	4 (11)	—
L3 AIS A ZPP	42 (21)	21 (24)	11 (18)	7 (18)	3 (37)
L3 AIS B, C, D	18 (9)	5 (6)	6 (10)	4 (11)	3 (37)
L4‐S1 AIS A	1 (1)	1 (1)	—	—	—
L4‐S1 AIS A ZPP	7 (4)	2 (2)	2 (3)	3 (8)	—
L4‐S1 AIS B, C, D	9 (5)	4 (4)	3 (5)	2 (5)	—
AIS E	8 (4)	6 (7)	1 (2)	1 (2)	—
Muscle function group (MF)					
MF 0	17 (9)	10 (11)	3 (5)	4 (10)	—
MF I	18 (9)	9 (10)	5 (8)	2 (5)	2 (25)
MF II	36 (18)	15 (17)	9 (15)	9 (24)	3 (38)
MF III	58 (30)	26 (29)	20 (33)	9 (24)	3 (37)
MF IV	33 (17)	13 (14)	11 (19)	9 (24)	—
MF V	34 (17)	17 (19)	12 (20)	5 (13)	—
Ambulatory function					
1	71 (37)	33 (37)	18 (30)	15 (39)	5 (64)
2	13 (6)	6 (7)	3 (5)	3 (8)	1 (12)
3	17 (9)	10 (11)	5 (8)	1 (3)	1 (12)
4	5 (2)	4 (4)	1 (2)	—	—
5	17 (9)	10 (11)	6 (10)	1 (3)	—
Do not walk	73 (37)	27 (30)	27 (45)	18 (47)	1 (12)
Transfer chair/wheelchair to bed					
Independent	166 (85)	74 (82)	52 (87)	32 (84)	8 (100)
With support	16 (8)	6 (7)	7 (11)	3 (8)	—
Lift	14 (7)	10 (11)	1 (2)	3 (8)	—
Orthoses					
Insole	27 (14)	12 (13)	7 (12)	8 (21)	—
SMO	2 (1)	1 (1)	1 (2)	—	—
AFO	41 (21)	22 (24)	11 (18)	6 (16)	2 (25)
KAFO	12 (5)	11 (12)	1 (2)	—	
Walking aids	25 (13)	8 (9)	8 (13)	6 (16)	3 (38)
Cane/Crutches	16 (8)	4 (4)	5 (8)	2 (5)	2 (28)
Walker	9 (5)	4 (4)	3 (5)	4 (11)	1 (13)
Wheelchair					
Manual	52 (27)	22 (24)	24 (40)	4 (11)	2 (28)
Powered	4 (2)	1 (1)	1 (2)	2 (5)	—
Manual and powered	67 (34)	32 (36)	18 (30)	16 (42)	1 (13)

Note

Number (*n*), American spinal injury association impairment scale (AIS), Zone of partial preservation (sensory or motor) (ZPP).

*Muscle function group (MF):* MF 0: with no loss of muscle strength. MF I: with weakness in foot intrinsic muscles and plantar flexors, grades 4–5. MF II: with foot plantar flexion grade 3, knee flexion grade 3, hip extension and/or hip abduction, grades 2–3. MF III: with hip flexion and knee extension, grades 4–5, knee flexion, grade 3, and only traces of hip extension, hip abduction, and below‐knee muscles. MF IV: no knee extension activity, hip flexion (grade ≤ 2), fair or good pelvic elevation. MF V: no muscle activity in the lower limbs, no pelvic elevation.

*Ambulatory function:* 1 Community ambulation; 2 Community ambulation, wheelchair use only for long distances outdoors; 3 Household ambulation, wheelchair outdoors; 4 Household ambulation, wheelchair both in‐ and outdoors; 5 Nonfunctional ambulation, ambulation during therapy. Wheelchair for mobility.

Supra‐malleolar orthosis (SMO), ankle–foot orthosis (AFO), free‐articulated knee–ankle–foot orthosis (KAFO).

### 3.4 Cognitive subtests

The participants performed one *SD* below the reference values for the general population on the cognitive assessments (Table 5, coding, block design, and FAS; presented as scores on a scale). Significant differences (*p* < .001) were seen between participants with and without HC in this regard. All individuals without HC performed within the reference values for the general population, albeit on the lower side of the spectrum. Subtests of cognitive characteristics are presented in Table 5.

**TABLE 5:** Cognitive subtests

	Total *n* = 153 HC, *n* = 98 non‐HC, *n* = 55	Age group 1 (18–30 years) *n* = 71 HC, *n* = 55 non‐HC, *n* = 16	Age group 2 (31–45 years) *n* = 49 HC, *n* = 35 non‐HC, *n* = 14	Age group 3 (46–60 years) *n* = 27 HC, *n* = 7 non‐HC, *n* = 20	A ge group 4 (≥61 years) *n* = 6 HC, *n* = 1 non‐HC, *n* = 5
Coding, mean (*SD*)	6.8 (3.1)	6.5 (2.7)	6.7 (2.8)	7.4 (4.2)	8.5 (4.0)
HC	5.6 (2.4)	5.7 (2.2)	5.9 (2.8)	3.7 (1.4)	5
Non‐HC	8.9 (3.1)	9.3 (2.3)	8.6 (1.7)	8.7 (4.1)	9.2 (4.1)
Block design mean (*SD*)	6.9 (2.7)	6.2 (2.5)	7.2 (2.5)	7.6 (3.3)	8 (3.0)
HC	6.1 (2.3)	5.8 (2.3)	6.9 (2.5)	4.9 (1.3)	5
Non‐HC	8.2 (2.9)	7.9 (2.8)	8.0 (2.6)	8.6 (3.2)	8.6 (2.9)
FAS^a^, mean (*SD*)	7.3 (3.7)	8.1 (3.5)	6.8 (3.7)	6 (4.0)	7.0 (3.4)
HC	6.7 (3.5)	7.4 (3.2)	6.3 (3.5)	2.7 (1.8)	3
Non‐HC	8.5 (3.8)	10.2 (3.5)	7.8 (4.0)	7.7 (3.8)	7.8 (3.1)

Note

Number (*n*), hydrocephalus (HC), standard Deviation (*SD*).

Reference value for the general population is 10 (*SD* = 3). The range of scores on the scale is 1–19 (mean = 10 and *SD* = 3).

^a^ To calculate the score on the FAS scale, results were first converted to *Z*‐values.

## 4 DISCUSSION

This study presents an overview of an almost total regional population of adults (196 of 219 enrolled ) with SB in Sweden, which has never been done before. The results demonstrate the multifaceted disabilities this cohort experiences, the interplay of medical, physical, cognitive, and social components in their everyday life.

Results show that individuals >46 years with SB had fewer associated conditions such as HC, latex allergy, and tethered cord symptoms. Individuals >46 years also who walked to a greater extent had better bladder, bowel, and cognitive functions than the participants <45 years. Taken together, participants >46 years had better prerequisites to live independently, had attained a higher level of education, and had participated more in society. There was a significantly higher prevalence of hydrocephalus and tethered cord symptoms among participants in 18–30 years, and significantly fewer had passed primary school. Persons in this group were born between 1985 and 1997, and consequently had grown up with improved treatments such as CIC (introduced in the 70s), shunt treatment for HC (introduced in the 60s), and magnetic resonance imaging (MRI, introduced in the 70s) assessments for other potential diagnoses. The reason why fewer passed primary school can only be speculative; one reason may be that those with more severe conditions had survived but found it hard to pass the core subjects.

Approximately half of the participants received municipal or state assistance. For many, this was not enough, so extra help from relatives was necessary. FIM measures the level of a person's independence and indicates how much assistance is required for the individual to carry out activities of daily living (Dodds et al., 1993). The mean FIM motor score in our cohort (in most cases a midlumbar spinal cord malformation) corresponded to FIM scores for persons with acquired spinal cord injury (SCI) on a cervical level (C8) (Hall, Cohen, Wright, Call, & Werner, 1999), indicating that persons with SB have more difficulty performing personal care activities. This notable difference is probably because SB involves the total central nervous system, not only the spinal cord. Almost half of the participants that received assistance with household activities and/or personal care were also dependent on reminders to manage their daily life. To our clinical experience, the relatives are often advising their adult children and as such, helping with compensating cognitive challenges to make daily life function for adults with SB. The situation for relatives who assist adults, with so‐called childhood disabilities, is an unexplored area that needs more focus (Hallberg & Hallberg, 2015).

One‐fifth of the participants was unemployed, and another fifth was in different educational programs, many due to unemployment. Only 22% of this population had a full‐time job; 37% of the participants worked either a part‐time or a full‐time job. This is lower than in a study of adults with SB (*n* = 72) in the United States, where 57% worked (Wagner et al., 2015). However, different social systems make it hard to compare results. A Norwegian study (Lidal et al., 2019) with a social system more like Sweden reported that 53% of their cohort (adults > 50 years) were employed, which corresponds with results in this study where 50% of participants > 46 years were employed (41% were retired). Social policies in Sweden have managed to integrate adults born with a disability into the regular educational system but have failed to offer them permanent work (Törnbom et al., 2011). Difficulties in finding an appropriate occupation are affected by the multifaceted problems of this cohort and negatively influence their living conditions and ability to structure their day. Lack of structure makes it harder to follow routines, such as CIC, an important aspect of health for this population.

Most participants reported problems with both urinary and faecal incontinences, and of those, one‐third reported daily urinary incontinence. Szymanski et al. (2016, 2017) have concluded that incontinence and specifically increasing amounts of urinary incontinence have a profound impact on health‐related quality of life (HRQOL) in persons with SB. Faecal incontinence impacts HRQOL regardless of frequency or amount (Szymanski et al., 2018).

Two‐thirds of the participants had a BMI higher than normal, and around one‐third were obese. However, using BMI in adults with SB may underestimate the true incidence of obesity by almost half (Liu et al., 2016), indicating that an even higher proportion of this cohort is obese. Adults with MMC have an increased risk of cardiovascular disease from multiple factors such as physical inactivity and obesity (Buffart et al., 2008). Overweight profoundly affects the ability to move and might affect independence. Overweight also increases the risk of pressure ulcers (Elsner & Gefen, 2008). The weight load on potential walking aids increases, and the person might need a wider wheelchair, increasing the risk of shoulder‐related problems and the need for a powered wheelchair and more help in daily life. Previous literature indicates an inactive lifestyle in persons with SB (Crytzer, Dicianno, & Kapoor, 2013). This in congruence with our results where only half of the cohort stated that they performed exercise with at least the moderate intensity required to achieve health‐related benefits (WHO, 2017b). Exercise has been found to contribute to other social benefits, such as being ‘part of a social context’ and ‘getting out of the house’ (Gabrielsson, Traav, & Cronqvist, 2015), which are important for psychological well‐being.

It is well known that HC is negatively associated with cognitive function and thereby potentially interferes with many aspects of daily life (Dennis et al., 2006; Hampton et al., 2011; Wetzel et al., 2018; Zabel et al., 2011). Our results show that especially participants with HC had considerably more impaired cognitive function than the reference values for the general population, particularly when psychomotor and executive functions, and, to some extent, verbal executive function and mental speed were assessed. Cognitive impairment complicates everyday life and makes it hard to handle life in a body that requires continuous attention (O'Hara & Holmbeck, 2013). Intellectual disability was found in 14% of the participants in this cohort, and this is comparable with a study of young adults with SB (mean age 20 years) where the corresponding number was 20% (Verhoef et al., 2004).

Earlier studies point at the importance of lifelong medical follow‐up by a multidisciplinary team for persons with SB (Lidal et al., 2019; Olsson et al., 2007; Roach, Short, & Saltzman, 2011; Webb, 2010), but our experience is that this does not solve all the problems. Many would benefit from a coach who helps identify needs and priorities and structure everyday life to prevent the associated conditions from occurring and to ensure that solutions to various problems are coordinated. Suggestions for future research are to focus on the growing adult population and development of sustainable follow‐up for all persons with SB. This should include development of social arenas for the importance of being in a context, for example meaningful occupation and the legal right to appropriate support on an individual level to enable a full life throughout the life span.

## 5 STRENGTHS AND LIMITATIONS

The type of SB presented in this study has been researched extensively in the patient medical records outside our clinic. The diagnosis in current adult medical records is specified as spina bifida with or without HC. In the future, with the national quality register and follow‐up program recently introduced in Sweden, the type of SB/MMC will be more precise and easier to find (Alriksson‐Schmidt et al., 2017) 2019. This will be an improvement since the term ‘spina bifida’ can be considered too ambiguous since other NTDs than MMC may also be included.

The strengths of this study include the large sample size and the extensive data collection by a multidisciplinary team, the few dropouts, and the broad age range of the adult participants. We are aware of a few individuals (<5) who have turned down offers to receive healthcare follow‐up at this specialized outpatient clinic. The national register (previously mentioned) will improve the ability to know how many adults with SB are there in the country (today no exact number exists) and to better follow them throughout the country.

It is a challenge to describe an adult population who live complex lives with SB without dwelling on all the details related to many specific areas. However, several scientific reports from this cohort will follow. The objective of this specific article was to describe a view of the adult population with this disability. The results are divided into age groups which did not have an equal number of participants; however, this was expected as there are not yet many adults >46  today with SB. There was no information on whether the participants who had employment received wage subsidies or not. Knowledge concerning wage subsidies would have given a more accurate employment rate. Since some of the participants had help from their assistance provider to answer the questions, due to cognitive impairment, this could have biased the answers.

## 6 CONCLUSIONS

This study demonstrated that adults with SB have a complex set of medical, physical, cognitive, and social needs that need to be addressed to improve their health and living conditions. Adults with SB have a high prevalence of urinary and faecal incontinence, pain, and overweight, which underlines that these issues need much attention during follow‐up. Today, persons who have survived to the age of >46 years had less complex medical conditions, and better physical and cognitive functions, and had attained a higher level of education, leading to better prerequisites for living independently and participating in society. The future generations of older adults may need more attention in many ways, since they at a younger age do have more complex medical conditions, lower physical and cognitive functions, and lower prerequisites for independent living and participation in society than those> 46 years today. This elucidates that adults with spina bifida need systematic follow‐up services and social support throughout life.

Increased knowledge allows targeted interventions and better care, thereby decreasing the risk for secondary conditions, reducing costs for society, and hopefully increasing the quality of life for persons with SB. This study provides an overall picture of the participants' health issues and living conditions. However, in‐depth research is needed to explore several subthemes such as the associations between physical and cognitive functions and experiences of living as an adult with SB.

## CONFLICT OF INTEREST

The authors declare no potential conflicts of interest with respect to the research, authorship, or publication of this article.

## AUTHOR CONTRIBUTION

MB, HG, DR, and ÅS conceived the idea for this study and designed it along with the other authors. Data collection was performed by MB, HG, GH, and DR. Authors MB, HG, GH, and ME conducted data analysis, while all authors (MB, HG, DR, GH, EF, CH, ME, ÅS) contributed to interpretation of data. MB and HG drafted the manuscript, and all authors revised the manuscript for important intellectual content and approved the final version. MB and HG contributed equally to this work.

### Peer Review

The peer review history for this article is available at https://publons.com/publon/10.1002/brb3.1736.

## Data Availability

The dataset generated during the current study is not publicly available due to Swedish and EU personal data legislation but is available from the corresponding author on reasonable request. Any sharing of data will be regulated via a data transfer and user agreement with the recipient.
